# RBM15 affects mitophagy in intracerebral hemorrhage by promoting E2F1 nuclear translocation via NXF1

**DOI:** 10.3389/fimmu.2026.1892295

**Published:** 2026-09-16

**Authors:** Jiwei Sun, Haoyu Yin, Jian Song, Ning Li, Jie Feng, Xuefeng Lu, Yi Han, Yesen Zhang

**Affiliations:** Department of Neurosurgery, The First Affiliated Hospital of Bengbu Medical University, Bengbu, China

**Keywords:** E2F transcription factor 1 (E2F1), HT22 cell, intracerebral hemorrhage (ICH), nuclear RNA transport factor 1 (NXF1), RBM15

## Abstract

Nuclear mRNA transport is essential for maintaining mRNA homeostasis and normal cellular functions; however, the mechanisms regulating the nuclear transport of E2F transcription factor 1 (*E2F1*) mRNA remain poorly understood. In this study, we show that the nuclear RNA transport factor 1 (NXF1) is upregulated in Hemin-treated HT22 cells and intracerebral hemorrhage (ICH) mice, suggesting its involvement in ICH pathogenesis. *NXF1* knockdown exerted a protective effect against Hemin-induced inflammatory responses in HT22 cells. Consistently, *NXF1* downregulation ameliorated neurological deficits, reduced cell death, and decreased cytokine levels *in vivo*. Mechanistically, NXF1-mediated neuroprotection was associated with mitophagy induction. Collectively, our findings provide novel insights into ICH progression and identify the RBM15/NXF1/E2F1 axis as a potential target for ICH intervention.

## Background

Intracerebral hemorrhage (ICH) is a life-threatening condition resulting from rupture of cerebral blood vessels (1–3). In recent years, considerable advances have been made in understanding the pathophysiology of ICH, as well as in improving its treatment and prevention (4, 5). However, the clinical outcomes remain poor. Therefore, it is necessary to understand the mechanisms underlying ICH.

Brain tissue injury and functional impairment following ICH involve complex pathophysiological processes. In addition to the primary mechanical damage caused by the hematoma, the associated inflammatory response is regarded as a pivotal factor that drives the progression of brain injury and influences patient prognosis (6). When the blood components extravasate into the brain parenchyma, they rapidly activate immune cells, thereby releasing large quantities of inflammatory mediators, including TNF-α, IL-6, and IL-1β (7). Therefore, inhibiting the inflammatory response is considered as a key therapeutic target for ICH.

m6A methylation is a crucial post-transcriptional mRNA modification, catalysed by METTL3, METTL16, and RBM15/RBM15B (8, 9). Abnormal m6A methylation is tightly correlated with the onset of several diseases, including non-small cell lung cancer (NSCLC) (10–12), cardiovascular disease (13), and ICH (14, 15). Previous studies demonstrated that m6A methyltransferase knockdown significantly alleviates ICH progression (14, 15). Further, as a newly discovered methyltransferase, RBM15 is involved in various cellular processes, but its exact role in the progression of ICH is still unclear (16–18). Our previous data showed that RBM15 is involved in ICH progression as it facilitates the m6A methylation of E2F1 mRNA (19). However, the molecular mechanism mediating E2F1 mRNA nuclear export remains to be fully elucidated.

Generally, mRNA nuclear export in eukaryotes is mediated by the nuclear RNA transport factor 1 (NXF1) and NTF2-like export protein 1 (NXT1), which are highly conserved from yeast to humans (20–23). These proteins bind to export-ready mRNA ribonucleoprotein complexes (mRNPs), facilitating their recognition and targeting to the nuclear pore complex (NPC). Once in the NPC, the NXF1-NXT1/p15 dimer (24–26) plays a key role in mRNA nuclear translocation, ensuring proper export of mRNA for translation and other cellular processes (27–29). Therefore, disorders of mRNA nuclear transport are closely associated with several diseases, including Alzheimer’s disease (AD), Huntington’s disease, and Frontotemporal Dementia (30–33). Previous studies have demonstrated that RBM15 interacts with NXF1 through their C-terminal regions. This interaction is crucial for enhancing the ability of NXF1 to bind mRNA (34–36). However, it remains unclear whether NXF1 is involved in the progression of ICH. Furthermore, the role of NXF1 in ICH and the importance of its interaction with RBM15 in the nuclear transport of E2F1 mRNA remains unknown. Therefore, the aim of this study was to investigate the mechanisms underlying nuclear export of E2F1 mRNA.

## Materials and methods

### ICH mouse model

C57/BL6 male mice (6-8 weeks old) were purchased from Huachuang Sino (Jiangsu, China) and raised under specific pathogen-free conditions for one week. The ICH model was established based on previous experimental methods (37, 38). Briefly, mice were randomly divided into four groups: Sham, ICH, ICH+sh-NC, and ICH+sh-NXF1. The basal ganglia were targeted at coordinates: 0.6 mm anterior, 2.0 mm to the left, and a depth of -3.2 mm. Using a microsyringe devoid of anticoagulants, approximately 40 μL of femoral artery blood was slowly aspirated. The injection pump parameters were set to 1 μL/min. At first, 10 μL of blood was infused over 10 min, and the needle was left in place for 5 min. Subsequently, the remaining 30 μL of blood was infused over 30 min. Finally, the needle was slowly withdrawn, the puncture site was sealed with bone wax, the skin incision was sutured, and the mouse was removed from the stereotaxic apparatus.

The mice in the sham group were injected with an equal volume of saline. The sh-NC and sh-NXF1 group mice were injected with the corresponding lentivirus (1x10^7^ TU) 3 days prior to constructing the ICH model. The mice were killed 3 days after establishment of the model.

### Brain tissue water content

On the third day after ICH, brain tissues were removed, weighed, and then dried at 95 °C for 24 h to obtain the dry weight. The formula for calculating the water content of the brain tissue was as follows: brain water content =(wet weight-dry weight)/wet weight x 100%.

### Apoptosis assay

The brain tissues were cut into 8-micron sections and stained with TUNEL (KCD-T1011) or Fluoro-Jade C (Solarbio, G3262) working solution. The sections were incubated with the respective working solution for 1 h at 37 °C. After incubation, the sections were stained with DAPI to label the nuclei. After washing with PBS, the results were observed under a fluorescence microscope (Olympus, BX53).

### Cell culture and transfection

HT22 cells (Pricella, CL-0697) were cultured in DMEM supplemented with 10% fetal bovine serum (FBS) at 37 °C under 5% CO_2_.

Establishment of the ICH cell model: HT22 cells were treated with 25 μmol/L of Hemin for 24 h. This treatment mimicked the pathological conditions of ICH *in vitro*. After Hemin treatment, cells were harvested for subsequent analysis.

For cell transfection: sh-RBM15, sh-NXF1, and their corresponding controls were transfected into HT22 cells using Lipo 2000 (Thermo, p11668019).

sh-NXF1-1: 5’-CGAGTAAGATACAATCCCTAT-3’; sh-NXF1-2: 5’-GCGAACGATTTCCCAAGTTAT-3’; sh-NXF1-3: 5’-GCGGGAATTAGACAAGATAAA-3’. sh-RBM15: 5’-AGGAACCTTGTGTCCTATTTA-3’. sh-E2F1: 5’-GATCTCTTTGACTGTGACTTT-3’.

### RNA-immunoprecipitation

HT22 cells were lysed using RIP lysis buffer, the cell lysate was then incubated with YTHDC1 or IgG antibody pre-bound to protein A/G magnetic beads at 4 °C. After washing with RIP wash buffer, RNA was extracted using TRIzol reagent, followed by reverse transcription of the RNA into cDNA. Finally, qPCR was employed to calculate the relative enrichment fold of E2F1.

### Western blot analysis

Total protein was extracted from HT22 cells or brain tissue using the RIPA lysis method. The antibodies used in this study were as follows: Rabbit anti-β-actin (Servicebio, GB11001), Rabbit anti-RBM15 (Proteintech, 10587-1-AP), Rabbit anti-NXF1 (Proteintech, 10328-1-AP), Rabbit anti-PINK1 (Proteintech, 23274-1-AP), Rabbit anti-Parkin (Proteintech, 14060-1-AP), Rabbit anti-LC3 (Proteintech, 14600-1-AP), Rabbit anti-Lamin A/C (Proteintech, 10298-1-AP), and goat anti-rabbit (Biosharp, BL003A).

Cytoplasmic and nuclear proteins were extracted using a stepwise lysis method (Beyotime, P0027). First, solution A was added to the cell pellet and mixed thoroughly, followed by the addition of solution B. After vortex shaking, the mixture was centrifuged at 4 °C for 5 min; the resulting supernatant constituted the cytoplasmic protein. Next, nuclear protein reagent was added to the remaining pellet, followed by vortex shaking and centrifugation at 4 °C for 10 min. The resulting supernatant contained the nuclear protein. The obtained protein samples were then subjected to western blot analysis using Lamin A/C as an internal reference standard for nuclear proteins.

### Co-immunoprecipitation analysis

HT22 cells were lysed using IP Lysis buffer containing proteinase inhibitors, and NXF1 and RBM15 antibodies were added to the lysate to capture NXF1 and RBM15. After an overnight incubation, prewashed protein A/G beads were added to the sample, and incubated for 4 h. The purified protein complexes were eluted and collected for further analysis.

### GST pull-down

For the GST pull-down assay, bacteria expressing GST and GST-RBM15 were coupled to GSH beads and incubated with NXF1 antibody overnight at 4 °C. After incubation, the GST-binding buffer was used to wash the complexes to remove any unbound material. The eluted proteins were subjected to western blot analysis to identify the interaction between RBM15 and NXF1.

### Quantitative real-time PCR

Total RNA was extracted from HT22 cells using TRIzol reagent. Reverse transcription was performed according to the protocol for the reverse transcription kit (KCD-M1003).

The primers used in this study were as follows: β-actin forward: 5’-GTGACGTTGACATCCGTAAAGA-3’; reverse: 5’-GCCGGACTCATCGTACTCC-3’. E2F1 forward: 5’-TGCAGAAACGGCGCATCTAT-3’; reverse: 5’-CCGCTTACCAATCCCCACC-3’.

### Mito-tracker and LC3 staining

Samples were incubated with Mito-Tracker solution (YEASEN, 40741ES50) in a dark environment at 37 °C for 30 min. Then, the samples were fixed with 4% PFA for 10 min and then incubated with the LC3 antibody (Abcam, ab192890) at 4 °C. After incubation with secondary antibodies, the results were observed under a fluorescence microscope.

### JC-1 staining

HT22 cells: Samples were incubated with JC-1 working solution (MCE, HY-K0601) in a dark environment at 37 °C for 30 min. After washing three times with PBS, the samples were incubated with DAPI solution to label the nucleus.

Brain tissues: After fresh brain tissue was subjected to freezing sectioning, the sections were incubated with JC-1 working solution (HEPENG BIOLOGICAL, HPBIO-JM196) at 37 °C for 20 min. Subsequently, the sections were incubated with DAPI to label the nuclei. After washing with PBS, the sections were observed under a fluorescence microscope.

### MitoSOX staining

The samples were incubated with MitoSox Red solution (YEASEN, 40778ES50) in dark at 37 °C for 10 min. After washing with PBS, an anti-fluorescence quenching sealing agent was added. Finally, samples were observed under a fluorescence microscope.

### Enzyme-linked immunosorbent assay

The supernatants of HT22 cells and blood from the ICH mice were harvested for ELISA. The following ELISA kits were used for detection: mouse NSE ELISA kit (Elabscience, E-EL-M0089), mouse IL-10 ELISA kit (Elabscience, E-EL-M0046c), mouse IL-6 ELISA kit (Elabscience, E-EL-M0044c), mouse TNF-α ELISA kit (Elabscience, E-EL-M3063), mouse TGF-β ELISA kit (RnDSystems, DY1679), mouse IL-1 ELISA kit (Elabscience, E-EL-M0037c).

### Fluorescence *in situ* hybridization and immunofluorescence

HT22 cells were incubated with a commercial E2F1 probe (Biotechne, 431971), and washed with SSC solution. After washing, the cells were incubated with Rabbit anti-NXF1 (1:200, proteintech, 10328-1-AP) or Rabbit anti-RBM15 (1:100, proteintech, 10587-1-AP) primary antibodies at 4 °C. Next, the FITC-labeled goat anti-rabbit secondary antibodies (1:200, Servicebio, GB22303) were added and incubated at 37 °C for 1 h. Finally, the samples were observed under a fluorescence microscope.

### STRING website

The STRING website (https://cn.string-db.org/) was used to explore the RBM15-interacting proteins. “RBM15” was entered in the search box, the term “Human” was selected as the species to obtain proteins that interact with RBM15.

### Statistical analysis

The data were analyzed using GraphPad Prism 9.0 and presented as mean ± SD. Student’s t-test or one-way ANOVA followed by the Tukey’s test was used to compare the differences between two or multiple groups. All results were obtained from three independent biological replicates. Statistical significance was set at *P* < 0.05.

## Results

### RBM15 interacts with NXF1 and regulates its expression levels

Our previous data demonstrated that RBM15 promotes ICH progression by regulating the m6A methylation of E2F1 mRNA. We therefore investigated the mechanism by which RBM15 regulates E2F1 mRNA expression. STRING was used to search for RBM15-interacting proteins (**Figure 1A**). Among the identified proteins, we focused on NXF1, a nuclear mRNA transporter factor known to play a critical role in nuclear mRNA transport (39–41). Previous studies have indicated that NXF1 is involved in RBM15-mediated nuclear mRNA transport (34, 35). Therefore, we selected NXF1 for subsequent experiments.

**Figure 1:**
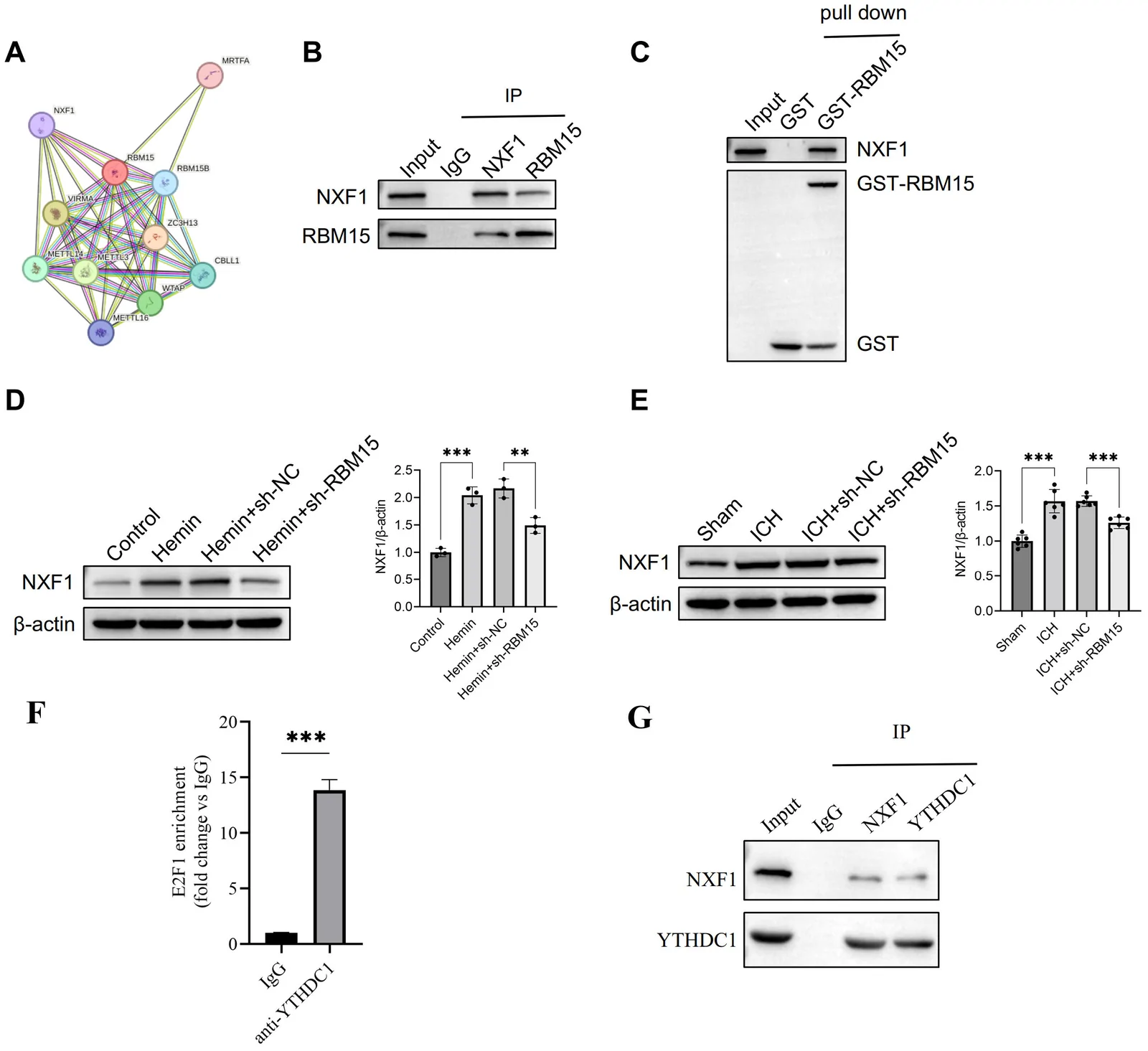
RBM15 interacts with NXF1 and regulates its expression levels. **(A)** The STRING website was used to predict the interacting proteins of RBM15. **(B)** Interaction between RBM15 and NXF1 was assessed via co-IP analysis. **(C)** GST pull-down assay was used to detect the interaction between RBM15 and NXF1. **(D)** Changes in NXF1 expression levels in HT22 cells. **(E)** Changes in NXF1 expression in ICH mice. **(F)** RIP-PCR was used to detect the interaction between YTHDC1 and E2F1 mRNA. **(G)** Interaction between YTHDC1 and NXF1 was assessed via co-IP analysis. **P < 0.01, ***P < 0.001.

To investigate whether RBM15 interacts with NXF1, we performed Co-IP and GST pull-down experiments. The Co-IP results demonstrated that RBM15 interacted with NXF1 (**Figure 1B**), and this interaction was further confirmed by the GST pull-down assay (**Figure 1C**). Collectively, these data suggest that RBM15 interacts with NXF1.

Next, we examined whether RBM15 affects the expression levels of NXF1. Western blotting results demonstrated that NXF1 was upregulated in Hemin-treated HT22 cells and ICH mice (**Figures 1D, E**). Notably, *RBM15* knockdown reduced the protein expression level of NXF1 (**Figures 1D, E**). Our data suggest that NXF1 is involved in ICH progression and may play a critical role in RBM15-mediated alleviation of ICH.

YTHDC1 acts as a m6A reader, is crucial for pre-mRNA splicing, mRNA nuclear export, and mRNA stabilization (42–44). To investigate the molecular mechanisms underlying E2F1 mRNA nuclear export, we performed RIP-PCR and Co-IP experiments. Our data showed that YTHDC1 interacts with both E2F1 mRNA and NXF1 (**Figures 1F, G**). These data provide preliminary evidence that YTHDC1 may be involved in E2F1 mRNA nuclear export.

### Downregulation of NXF1 attenuates the inflammatory response in Hemin-treated HT22 cells

Next, we investigated whether *NXF1* knockdown exerts a regulatory effect on the inflammatory response. For this purpose, we knocked down *NXF1* in HT22 cells. qPCR results showed that compared to the sh-NC group, the sh-NXF1-2 group exhibited the lowest expression level of NXF1 mRNA (**Figure 2A**). Western blotting further showed that *NXF1* knockdown significantly reduced its protein expression levels (**Figure 2B**). Therefore, we selected this sequence for subsequent experiments.

**Figure 2:**
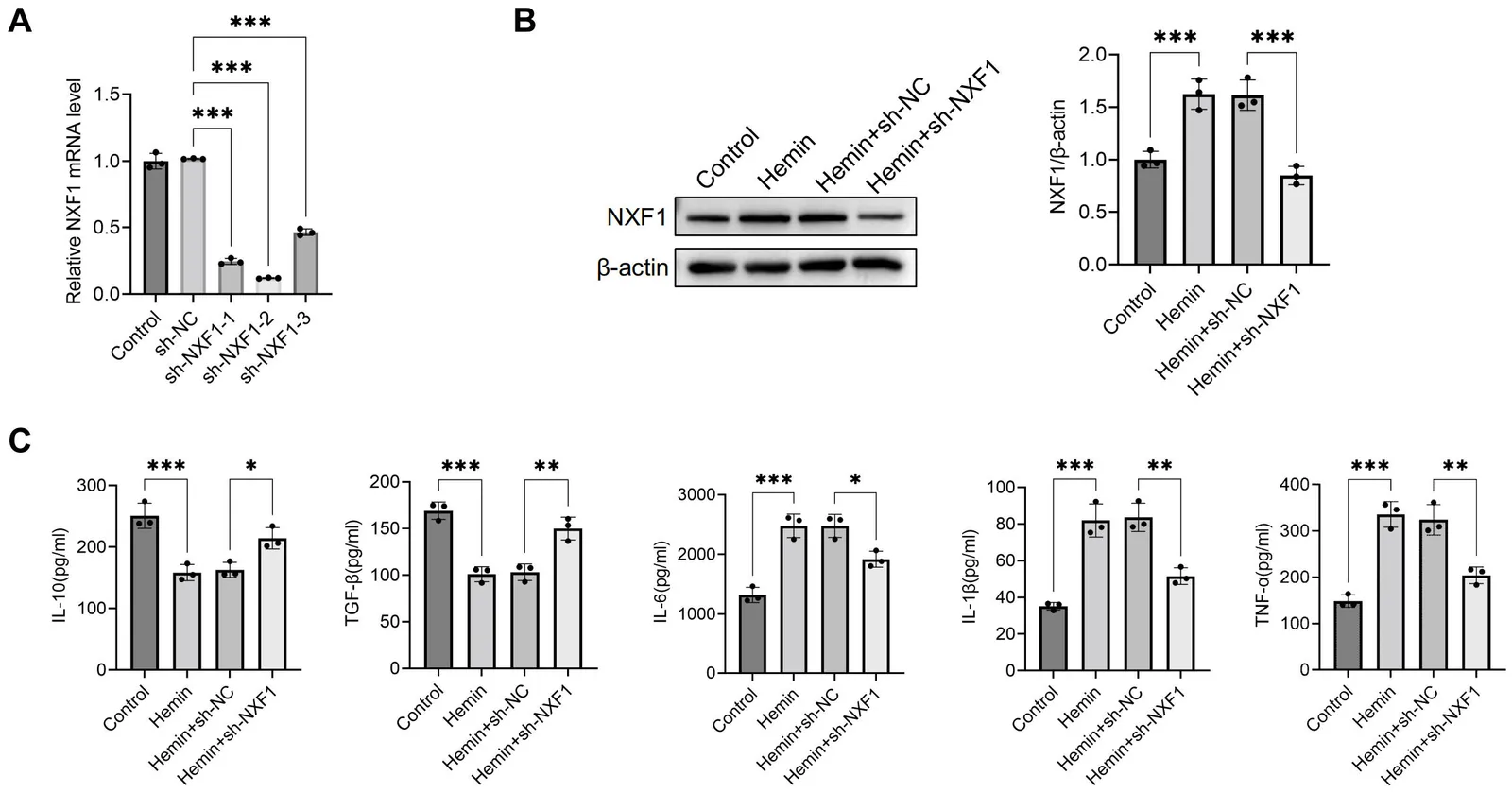
NXF1 knockdown attenuates inflammatory response *in vitro.* **(A)** Expression level of *NXF1* mRNA was assessed by qPCR. **(B)** Protein expression level of NXF1 was detected by Western blotting. **(C)** Cytokine levels were measured using ELISA. *P < 0.05; **P < 0.01; ***P < 0.001.

Additionally, the levels of anti-inflammatory factors, including IL-10 and TGF-β, were significantly increased, whereas the levels of pro-inflammatory factors, including IL-6, IL-1β, and TNF-α, were decreased after *NXF1* knockdown (**Figure 2C**). These data suggest that NXF1 knockdown-mediated suppression of the inflammatory response may exert a protective effect against Hemin-induced HT22 cell injury.

### Downregulation of NXF1 alleviates brain injury in ICH mice

To confirm whether NXF1 has a similar regulatory effect on ICH *in vivo*, we constructed an ICH mouse model. The results showed that, compared to the sham group, the ICH group exhibited a larger lesion volume (**Figure 3A**), elevated NXF1 protein expression levels (**Figure 3B**), and increased hematoma size (**Figure 3C**). Additionally, there was a significant increase in the heart rate, blood pressure, blood glucose levels, brain water content, and mNSS score (**Figure 3D**). Furthermore, there was a significant increase in cell death (**Figures 3E, F**), and increased levels of inflammatory factors and NSE (**Figure 3G**). Taken together, these data suggest that we successfully established an ICH mouse model that effectively recapitulates the key pathological features of ICH in humans.

**Figure 3:**
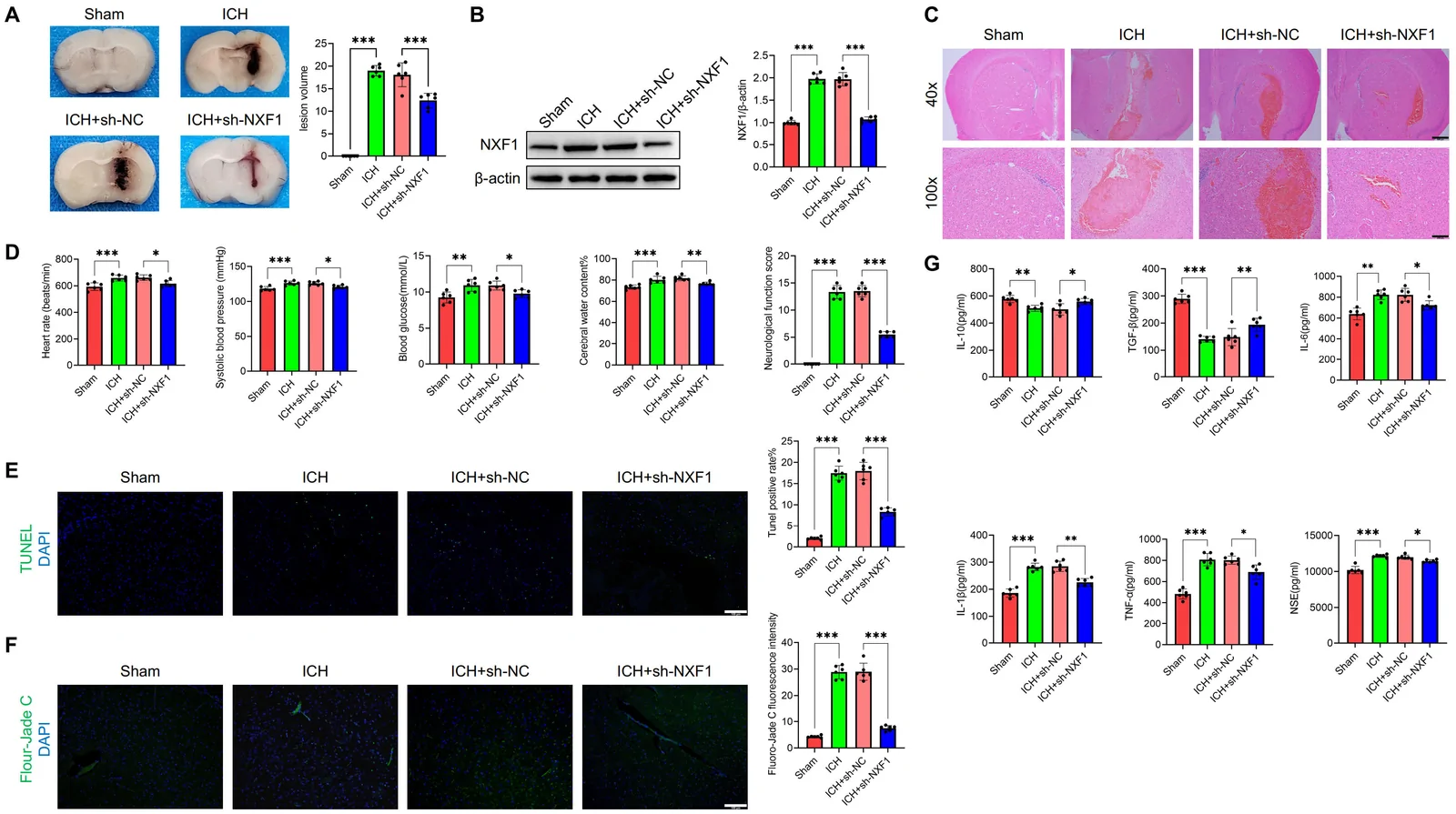
NXF1 down-regulation improves the pathological changes in ICH mice. **(A)** Changes in lesion volume after NXF1 downregulation in ICH mice. **(B)** Western blotting was used to detect the expression levels of NXF1 in brain tissue. **(C)** HE staining was used to detect hematomas in ICH mice. Scale bar: 500 μm (40x), 200 μm (100x). **(D)** Changes in heart rate, blood pressure, blood glucose level, brain water content, and mNSS in ICH mice. **(E)** TUNEL staining was used to detect cell apoptosis. Scale bar: 100 μm (200x). **(F)** FJ-C staining was used to detect apoptosis in neuronal cells. Scale bar: 100 μm (200x). **(G)** ELISA kits were used to detect the content of IL-6, IL-1β, TNF-α, IL-10, TGF-β, and NSE. *P < 0.05; **P < 0.01; ***P < 0.001.

Next, we used a lentivirus to interfere with NXF1 expression in ICH mice. The results showed that compared to the ICH+sh-NC group, the ICH+sh-NXF1 group showed a significant decrease in NXF1 expression levels (**Figure 3B**), a smaller lesion volume (**Figure 3A**), and a reduced hematoma size (**Figure 3C**). Additionally, there was a notable reduction in the heart rate, blood pressure, blood glucose levels, brain water content, and mNSS score (**Figure 3D**), indicating improved physiological conditions. Importantly, the number of TUNEL-positive cells (**Figure 3E**) and the fluorescence intensity of FJ-C were significantly reduced after NXF1 downregulation (**Figure 3F**), suggesting a decrease in cell death and neuronal injury. Moreover, ELISA results revealed an increase in the IL-10 and TGF-β levels, whereas the levels of IL-6, IL-1β, TNF-α, and NSE were reduced (**Figure 3G**). These data suggest that NXF1 downregulation exerts neuroprotective effects in mice with ICH.

### NXF1 knockdown promotes mitophagy during ICH progression

Next, we investigated the molecular mechanism by which NXF1 downregulation alleviates ICH progression. Since previous studies have reported that mitophagy is involved in the progression of ICH (45, 46), and we have demonstrated that the loss of RBM15 significantly alleviated ICH progression by activating mitophagy, we sought to explore the potential interaction between NXF1 downregulation and mitophagy.

We first investigated whether mitophagy is involved in ICH progression, both *in vitro* and *in vivo*. The results of MitoTracker and LC3 staining showed that LC3 expression was increased in both Hemin-treated HT22 cells and ICH mice (**Figures 4A, E**). JC-1 staining showed a marked decrease in the mitochondrial membrane potential of both Hemin-treated HT22 cells and ICH mice (**Figures 4B, F**). MitoSOX staining demonstrated a significant increase in mitochondrial ROS levels in both Hemin-treated HT22 cells and ICH mice (**Figures 4C, G**). Furthermore, the expression levels of Parkin, PINK1, and LC3 II were significantly upregulated in both Hemin-treated HT22 cells and ICH mice (**Figures 4D, H**). These findings suggest that mitophagy is involved in the progression of ICH.

**Figure 4:**
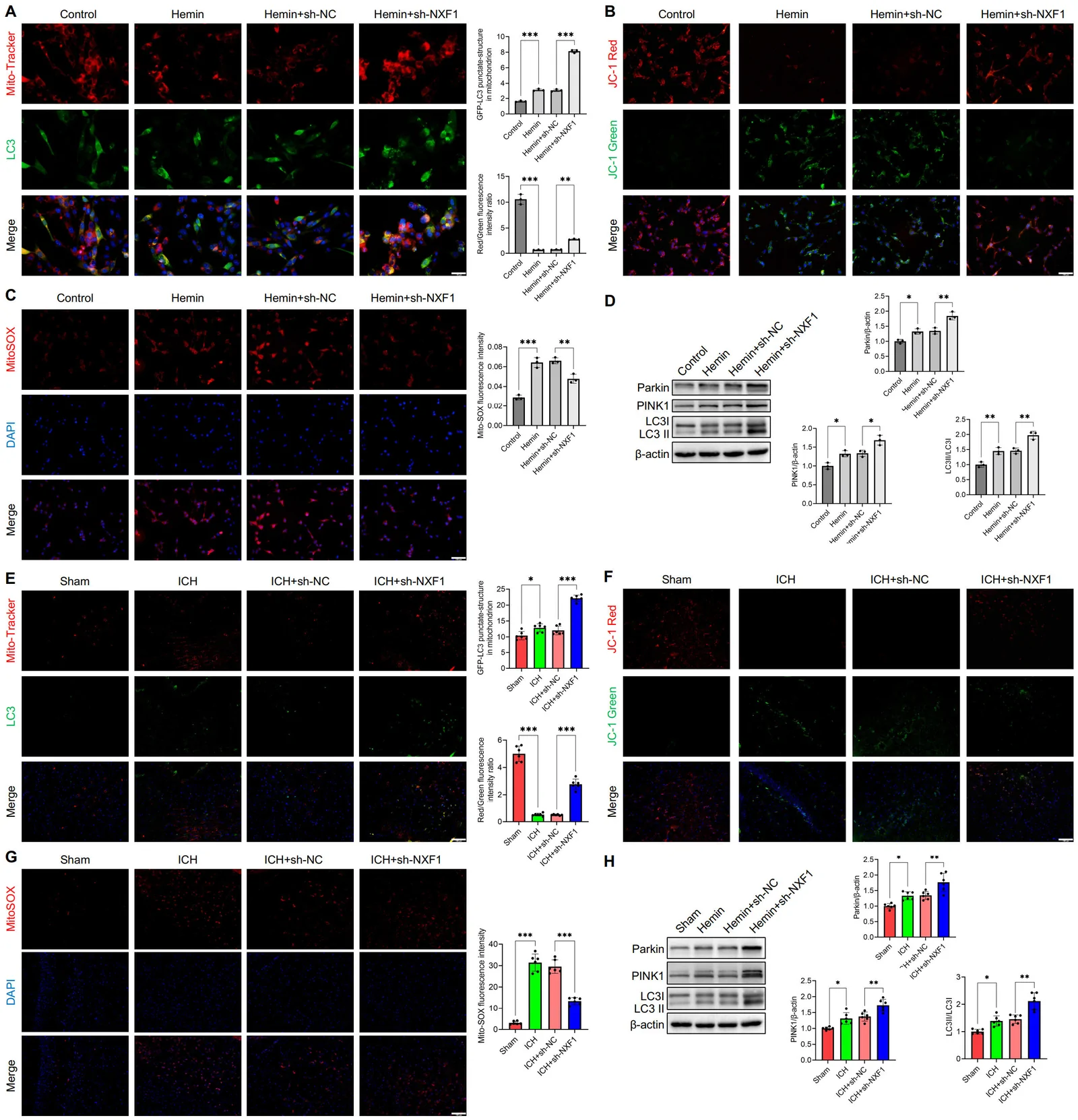
NXF1 down-regulation activates mitophagy both *in vitro* and *in vivo.* **(A)** Changes in mitophagy following *NXF1* knockdown *in vitro*. Scale bar: 50 μm (400x). **(B)** Changes in mitochondrial membrane potential after transfection. Scale bar: 100 μm (200x). **(C)** Changes in mitochondrial ROS levels after transfection. Scale bar: 100 μm (200x). **(D)** Changes in Parkin, PINK1, LC3I, and LC3 II protein expression levels after cell transfection. **(E)** Changes in mitophagy after NXF1 knockdown *in vivo*. Scale bar: 100 μm (200x). **(F)** Changes in mitochondrial membrane potential after NXF1 knockdown *in vivo*. Scale bar: 100 μm (200x). **(G)** Mitochondrial ROS levels were assessed using MitoSOX staining after NXF1 knockdown *in vivo*. Scale bar: 100 μm (200x). **(H)** The expression levels of Parkin, PINK1, LC3I, and LC3 II *in vivo* were analyzed by western blotting. *P < 0.05; **P < 0.01; ***P < 0.001.

Next, we downregulated *NXF1* expression and examined its effects on mitophagy. The results showed that *NXF1* downregulation further activated mitophagy, as evidenced by increased LC3 expression levels in the mitochondria (**Figures 4A, E**), enhanced mitochondrial membrane potential (**Figures 4B, F**), reduced mitochondrial ROS levels (**Figures 4C, G**), and increased expression levels of Parkin, PINK1, and LC3 II (**Figures 4D, H**). These data suggest that *NXF1* downregulation activates mitophagy, which may contribute to slowing ICH progression.

### RBM15 regulates E2F1 mRNA nuclear transport through NXF1

Previously, we have shown that RBM15 interacts with E2F1 mRNA and promotes its m6A modification. Furthermore, RBM15 interacts with NXF1, a nuclear mRNA transport factor. Based on these findings, we hypothesized that the nuclear transport of E2F1 mRNA depends on either RBM15 or NXF1.

To validate this hypothesis, we performed FISH and IF assays. The results showed that E2F1 mRNA was predominantly expressed in the nucleus, and NXF1 and RBM15 are expressed both in the nucleus and the cytoplasm (**Figures 5A, B**; **Supplementary Figure 1**). Next, we investigated whether NXF1 regulates E2F1 mRNA nuclear transport by knocking down *NXF1* in Hemin-treated HT22 cells. Compared to the control group, the nuclear-to-cytoplasmic ratio of E2F1 mRNA was reduced in the Hemin-treated group; however, knockdown of *NXF1* reversed this phenomenon (**Figure 5C**). qPCR results further corroborated these observations (**Figure 5D**), demonstrating that NXF1 is involved in the nuclear export of E2F1 mRNA.

**Figure 5:**
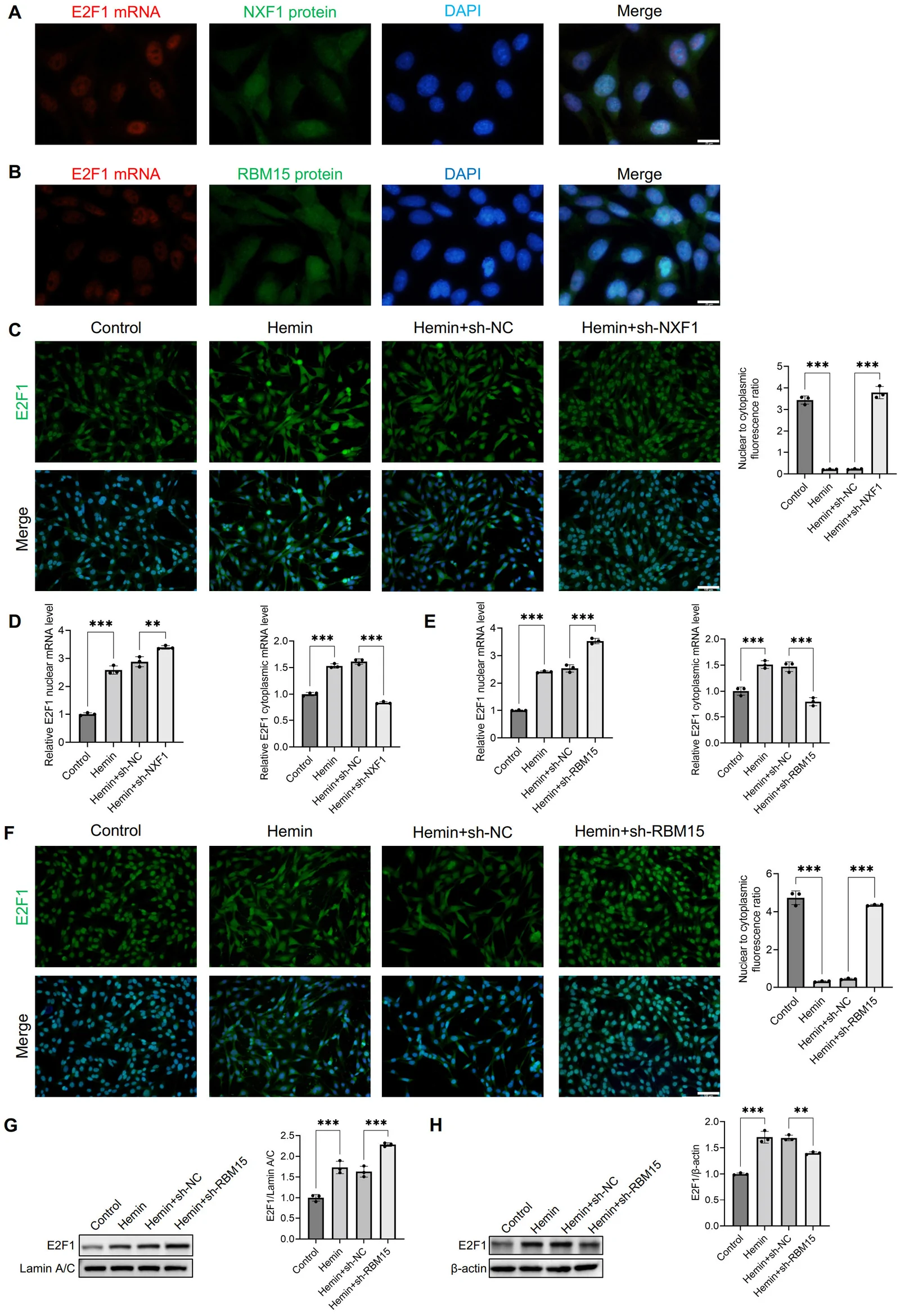
E2F1 mRNA nuclear transport was affected by RBM15 and NXF1. **(A)** FISH and IF were used to detect the interaction between E2F1 mRNA and NXF1 proteins. Scale bar: 25 μm (800x). **(B)** FISH and IF were used to detect the interaction between E2F1 mRNA and RBM15 proteins. Scale bar: 25 μm (800x). **(C)** Distribution of E2F1 mRNA in the cytoplasm and nucleus was detected by FISH after *NXF1* knockdown *in vitro*. Scale bar: 100 μm (200x). **(D)** qPCR was performed to detect *E2F1* mRNA expression in the cytoplasm or nucleus after *NXF1* knockdown *in vitro*. **(E)** qPCR was used to detect the E2F1 mRNA expression levels in the cytoplasm or nucleus after *RBM15* knockdown in HT22 cells. **(F)** FISH was used to detect the distribution of E2F1 mRNA in the cytoplasm and nucleus after RBM15 knockdown in HT22 cells. Scale bar: 100 μm (200x). **(G)** Western blotting was performed to evaluate the expression level of E2F1 in the nucleus after RBM15 knockdown. **(H)** The expression levels of E2F1 were detected in the cytoplasm after RBM15 knockdown. **P < 0.01; ***P < 0.001.

Next, we verified whether RBM15 has a regulatory effect on E2F1 mRNA nuclear transport. *RBM15* knockdown in Hemin-treated HT22 cells was performed. The results showed that *RBM15* knockdown significantly increased the nuclear-to-cytoplasmic ratio and reduced the cytoplasmic *E2F1* mRNA expression (**Figurea 5E, F**). Western blot results further confirmed these findings (**Figures 5G, H**). These data suggest that the downregulation of *RBM15* activates mitophagy by regulating the NXF1-mediated nuclear export of E2F1 mRNA, thereby alleviating ICH progression.

## Discussion

In this study, we identified a novel regulatory axis involving RBM15, NXF1, and E2F1 in ICH pathogenesis. Our data indicate that RBM15 positively regulates NXF1 expression, and that NXF1 downregulation exerts neuroprotective effects by mitigating the inflammatory response in Hemin-treated HT22 cells and ameliorating brain injury in ICH mice. Mechanistically, the neuroprotection mediated by *NXF1* down-regulation is associated with mitophagy activation, which likely contributes to the suppression of ICH progression. Furthermore, we found that RBM15 and NXF1 cooperatively regulate the nuclear transport of E2F1 mRNA. Collectively, our findings highlight the critical role of the RBM15/NXF1/E2F1 axis in ICH pathology and suggest that targeting this axis may represent a promising therapeutic strategy for ICH treatment.

Given the fundamental role of nuclear mRNA export in gene expression, dysregulation of the pathway is associated with various physiological and pathological defects (47). m6A methylation is a crucial step for binding to nuclear export factors such as NXF1 (48, 49). NXF1 is a nuclear receptor that is essential for nuclear mRNA transport. m6A methylated SRSF3 mRNA, regulated by the METTL3/METTL14 complex, showed higher binding efficiency with NXF1, thereby promoting its nuclear transport (50). Methylated FL mRNA interacts more effectively with NXF1 and is subsequently transported to the cytoplasm for further translation (51, 52). Consistent with these findings, our data suggest that NXF1 is essential for E2F1 mRNA nuclear transport. In Hemin-treated HT22 cells, E2F1 was predominantly located in the cytoplasm, and disturbed mitochondrial function, whereas knockdown of *NXF1* significantly decreased E2F1 mRNA levels in the cytoplasm and increased E2F1 mRNA levels in the nucleus. In addition to NXF1, we observed decreased expression of E2F1 mRNA in the cytoplasm in RBM15-deficient expression conditions. This phenotype may be due to insufficient m6A methylation of E2F1 mRNA or a reduction in NXF1 expression, which weakens the nuclear transport of E2F1 mRNA. Therefore, exploring whether NXF1 overexpression accelerates ICH progression by increasing E2F1 mRNA expression in the cytoplasm is crucial for understanding its underlying mechanism.

NXF1 acts as a nuclear transport factor and is closely associated with cellular functions. The NXF1/ALYREF complex is necessary for the development of BRC (53). In amyotrophic lateral sclerosis (ALS), excessive amounts of monomeric TAR DNA-binding protein-43 (TDP-43) are exported from the nucleus in an NXF1-dependent manner, leading to its mislocalization in the cytoplasm. This results in the phosphorylation and aggregation of TDP-43, ultimately triggering irreversible pathological changes associated with ALS (54). Previous data have revealed that RBM15 interacts with NXF1 and promotes the nuclear transport of mRNA (34). Consistent with previous studies, our results demonstrate an interaction between RBM15 and NXF1. Furthermore, the expression levels of NXF1 were elevated in Hemin-treated HT22 cells and ICH mice, and NXF1 expression was regulated by RBM15. Deletion of NXF1 exerts a protective effect following ICH injury, as NXF1 deficiency enhances cell survival, reduces cell death, improves neurological function, and decreases the inflammatory response. These findings suggest that NXF1 plays a determining role in the pathophysiology of ICH, and that targeting NXF1 may be a potential therapeutic strategy for improving outcomes following this type of brain injury.

Research on the relationship between NXF1 and mitochondria is limited. After the activation of PGC-1α target genes, NXF1 promotes mRNA nuclear export, thereby affecting mitochondrial homeostasis and metabolism (55). In the present study, we found that NXF1 affects mitochondrial homeostasis by promoting the nuclear transport of E2F1 mRNA. The deficiency of NXF1 in HT22 cells and ICH mice improves mitochondrial function by activating mitophagy, reducing ROS levels, and increasing the mitochondrial membrane potential.

This study has certain limitations. First, our data demonstrated that *NXF1* knockdown significantly attenuated the inflammatory response. As an effector factor of NXF1, E2F1 exerts regulatory effects on the NF-κB pathway (**Supplementary Figure 2**). However, whether other inflammation-related signaling pathways, such as MAPK and JAK-STAT, are involved in the inflammation response mediated by the NXF1/E2F1 axis requires exploration. Second, although our data demonstrate that RBM15 positively regulates the expression of NXF1, it remains to be determined whether this effect is mediated by its m6A methyltransferase activity or through an m6A-independent mechanism (such as protein–protein interactions or alternative splicing). Given that previous studies have reported the m6A-independent functions of RBM15, including its role in pre-mRNA splicing, we hypothesize that both mechanisms may be operative. Utilizing RBM15 catalytically inactivated mutants will be crucial for elucidating these possibilities. Third, the molecular mechanism underlying E2F1 mRNA nuclear transport requires investigation. Finally, the mechanism by which knockdown of *NXF1* reduced the hematoma volume, has not been resolved and may be attributed to inhibited secondary bleeding or enhanced hematoma clearance.

## Data Availability

The original contributions presented in the study are included in the article/**Supplementary Material**, further inquiries can be directed to the corresponding authors.

## Supplementary material

Supplementary Figure 1 The negative controls for FISH and IF experiments. Scale bars: 25 μm (800x).

Supplementary Figure 2 E2F1 regulates the NF-κB pathway in HT22 cells treated with Hemin.
